# Ultrabroadband Optical Diffraction Tomography

**DOI:** 10.1021/acsphotonics.4c00797

**Published:** 2024-08-27

**Authors:** Martin Hörmann, Franco V. A. Camargo, Niek F. van Hulst, Giulio Cerullo, Matz Liebel

**Affiliations:** † Dipartimento di Fisica, Politecnico di Milano, Piazza L. da Vinci 32, Milano 20133, Italy; ‡ Istituto di Fotonica e Nanotecnologie-CNR, Piazza L. da Vinci 32, Milano 20133, Italy; § ICFO − Institut de Ciencies Fotoniques, The Barcelona Institute of Science and Technology, Av. Carl Friedrich Gauss, 3, Castelldefels - Barcelona 08860, Spain; ∥ ICREA − Institució Catalana de Recerca i Estudis Avançats, Passeig Lluís Companys 23, Barcelona 08010, Spain; ⊥ Department of Physics and Astronomy, Vrije Universiteit Amsterdam, De Boelelaan 1081, Amsterdam, HV 1081, The Netherlands

**Keywords:** tomography, 3D imaging, broadband imaging, nanophotonics, Fourier transform spectroscopy, hyperspectral

## Abstract

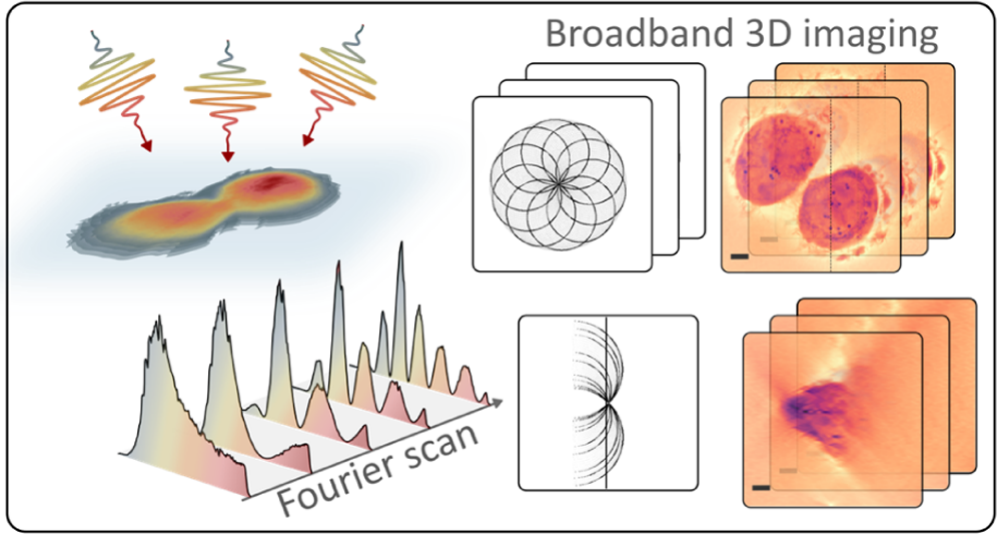

Optical diffraction tomography (ODT) is a powerful noninvasive 3D imaging technique, but its combination with broadband light sources is difficult. In this study, we introduce ultrabroadband ODT, covering over 150 nm of visible spectral bandwidth with a lateral spatial resolution of 150 nm. Our work addresses a critical experimental gap by enabling the measurement of broadband refractive index changes in 3D samples, crucial information that is difficult to assess with existing methodologies. We present broadband, spectrally resolved ODT images of HeLa cells, obtained via pulse-shaping-based Fourier transform spectroscopy. The spectral observations enabled by ultrabroadband ODT, combined with material-dependent refractive index responses, allow for precise three-dimensional identification of nanoparticles within cellular structures. Our work represents a crucial step toward time and spectrally resolved tomography of complex 3D structures with implications for life and materials science applications.

## 1 Introduction

Time-resolved spectroscopy studies the dynamics of light-induced processes, with applications ranging from fundamental photophysical processes over protein dynamics to complex devices and even single molecules.^1−3^ It is a very active field of research, as the ever-changing sample and material landscape calls for continuous innovation. Especially spatially resolved measurements have a tremendous impact on our understanding of, for example, nanoscale devices and biological materials. The combination of transient absorption spectroscopy with microscopy allows studying complex structure–function relationships with micro- to nanometre spatial and femtosecond temporal resolution. Examples are heat and carrier transport dynamics where exciting phenomena such as ballistic and hydrodynamic transport regimes have been uncovered.^4−6^ In parallel to the spectroscopic developments, spatially resolved pump–probe imaging, in the form of photothermal and phototransient approaches, is increasingly being used as a powerful imaging modality where pump-induced signals serve as a label-free means of contrast.^7−13^

**Figure 1:**
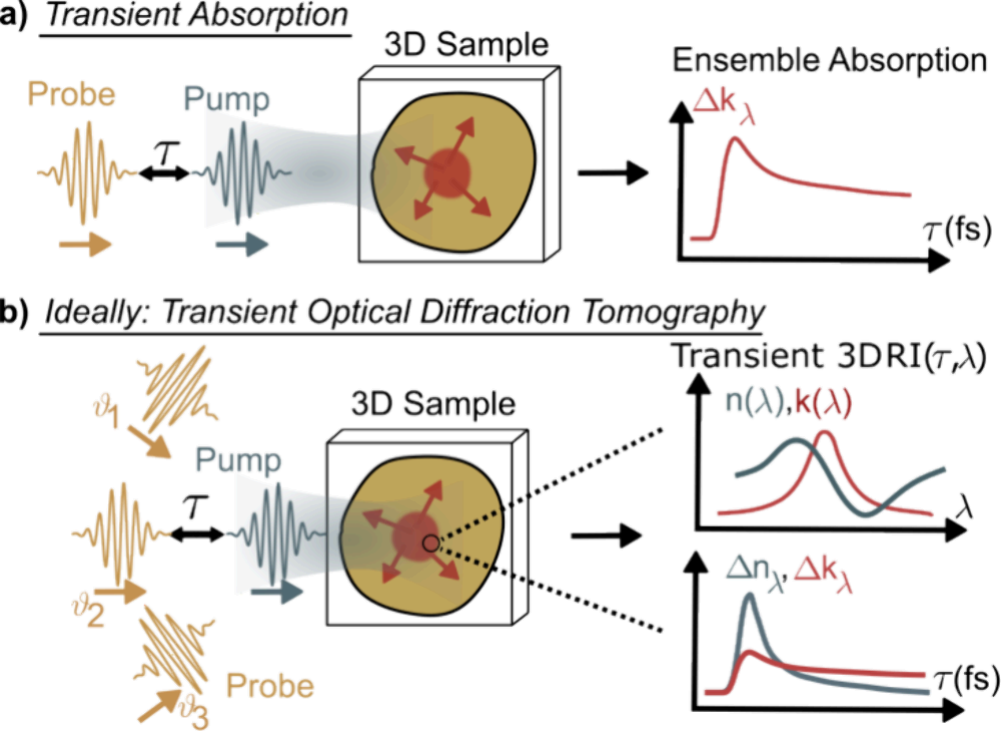
Toward 3D transient absorption tomography. (a) Conventional transient absorption measures time-dependent absorption changes of a spatial ensemble-average. (b) Optical diffraction tomography acquires an image stack at many different illumination, or probe, *k*-vectors to reveal the 3D complex RI distribution of a structured sample. A combination with transient excitation would enable full characterization of the spatiotemporal response of any sample of interest.

Most experiments provide spatially projected and often ensemble-averaged absorption changes (Figure 1a). However, samples are often three-dimensional and so is temporal information flow. In other words, existing techniques are unable to access the complex parameter space describing real samples, composed of spatial, temporal, and spectral degrees of freedom. A technique that goes beyond the state-of-the-art would be highly desirable. Ideally, one would like to directly access the temporal evolution of the spectrally resolved complex refractive index (RI) and, further, connect this information with the underlying 3D nanostructure of the system of interest. Important steps toward realizing this vision have been made,^6,13−15^ but a framework that enables transient complex RI spectroscopy with ultrabroadband/short pulses and nanometric spatial resolution on crowded 3D samples is still lacking. This shortcoming might, at first, be surprising as techniques such as optical diffraction tomography (ODT) readily provide 3D volumetric RI profiles, and even insight into the imaginary part of the RI, via computational synthesis based on multiple sample-projections.^16−21^ Combined with an excitation pulse, transient ODT should therefore be in reach (Figure 1b).

A major hurdle toward realizing transient measurements with ODT is the broad spectral bandwidth necessary to enable ultrafast observations whose temporal resolution is directly dictated by the pump and the probe pulse durations. ODT requires knowledge of both the phase and the amplitude of all sample projections, which is typically obtained holographically with spectrally narrow light. Nonholographic methods exist using intensity-only images^22−24^ but those rely on computational postprocessing that indirectly accesses the wave character of electric fields. Even though important steps toward increasing the bandwidth in holographic imaging have been taken, illumination bandwidths rarely exceed 10 nm,^12−14,25−27^ even for spectrally resolved ODT which requires wavelength sweeping.^28−30^ This is a problem since modern ultrafast transient absorption spectroscopy uses intrinsically ultrabroadband light pulses. Experimentally, it is challenging to combine ODT with ultrafast optics as the necessary broadband pulses exhibit extremely short temporal coherence lengths, far below 10 μm. Precise wavefront matching and ensuring correct temporal overlaps over large fields of observation and the entire wavelength range of a broadband pulse is highly nontrivial. The need to recover the spectral information on interest from images acquired in a color-blind fashion further complicates the experiment.

Here, we implement and validate ultrabroadband ODT as a first step toward true 3D transient absorption microscopy or phototransient imaging. Using off-axis holography together with high numerical aperture (NA)-based angle scanning, we perform ODT using pulses with >150 nm bandwidths, supporting durations of approximately 5 fs. We further provide, and validate, a strategy that allows recovering spectral information based on Fourier transform spectroscopy. Our work bridges the gap between three-dimensional imaging, via ODT, with ultrabroadband pulses exhibiting transform limited durations in the <10 fs range. Integrating our strategy with precompressed pump and probe pulses, using existing solutions,^12,13,31^ has the potential to directly access ultrafast 3D observations. More generally, we show that broadband light sources, with coherence lengths of a few micrometers, are compatible with interferometric wide-field imaging, such as off-axis holography, as long as spectral dispersion and path length are thoroughly accounted for. We demonstrate these capabilities by performing ODT—a challenging technique that requires varying illumination angles, which further complicates broadband interferometry. Thus, the presented principles and approaches are directly applicable to all imaging techniques that require exploiting short pulses and benefit from interferometric, or heterodyne, detection such as stimulated Raman scattering^32,33^ or photothermal imaging.^7−13,32^

The paper is organized as follows: we first give a detailed description of the experimental setup, addressing pitfalls, problems, and solutions when using broadband pulses for holographic imaging. We then discuss the general data-processing workflow, from raw holograms to spectrally resolved 3D RI representation. Following these experimental aspects, we validate that spectral interference over the entire bandwidth is achieved. We then present proof-of-principle spectrally resolved tomograms of HeLa cells. Finally, we conclude by performing two-color, spectrally multiplexed, ODT for nanoparticle identification in complex biological samples.

## 2 Methods

Spectrally resolved, ultrabroadband, ODT requires (i) a dedicated experimental setup suitable for temporally ultrashort light, (ii) a means of recovering spectral information, and (iii) computational processing to recover the spectrally resolved 3D information. In the following, we provide detailed information addressing these three key aspects.

### 2.1 Optical Diffraction Tomography via Broadband Off-Axis Holography

**Figure 2:**
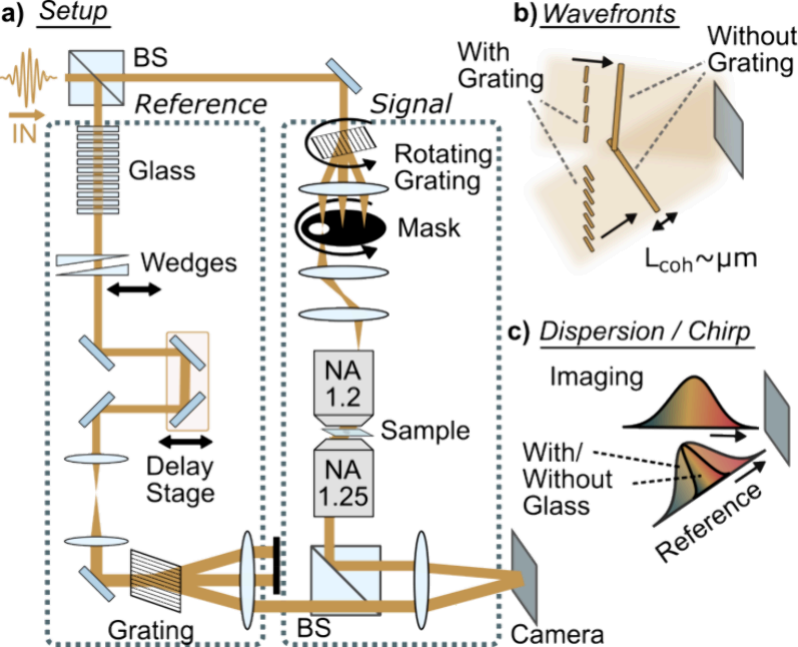
Broadband ODT setup and experimental considerations. (a) Supercontinuum-based broadband high-NA ODT setup relying on angle-scanning via grating rotation; BS: beam splitter. (b) Gratings in the sample and reference arms ensure parallel wavefronts and hence optimum interference. (c) Dispersion matching is necessary to ensure broadband interference.

Figure 2a depicts the ultrabroadband ODT imaging system based on the 480–670 nm output of a supercontinuum laser (*SuperK EXTREME*, *NKT Photonics*), mimicking the spectral range of typical Yb-laser pumped broadband noncollinear optical parametric amplifiers.^34,35^ The light enters a Mach–Zehnder interferometer that we conceptually divided into a signal and a reference arm to facilitate the discussion. Finally, a camera (*Q-2HFW, Adimec*) records the interference between the fields passing through the respective interferometric arms.

In more detail, the signal path contains a transmission microscope composed of an NA = 1.2 (*Olympus UPLSAPO60XW*, 60×, water immersion) and an NA = 1.25 (*Olympus UPLSAPO40XS*, 40×, silicone immersion) objective for illumination and collection, respectively. An imaging system composed of three lenses with effective focal lengths of 100, 100, and 240 mm (visible achromats, *Thorlabs*), and the illumination objective conjugates a rotating grating (Ronchi type, 20 grooves/mm, *Edmund Optics*; motorized rotation stage *PRM1Z8, Thorlabs*) with the sample plane. A hard aperture, placed in a Fourier plane of the grating and rotated by a second identical rotation stage, blocks all but the first diffraction order and allows interrogating the sample at precisely defined and adjustable, wave-vectors. The second microscope objective collects all the transmitted light, and a 500 mm lens (visible achromat, *Thorlabs*) forms an image of the sample on the camera.

The reference arm contains bulk material and a pair of 2° fused silica wedges (*LAYERTEC GmbH*) for precise chirp management. A translation stage matches the path length difference between the two interferometer arms. A 10× telescope expands the beam and eliminates minor wavefront curvature mismatch between signal and reference. A grating (19.5 grooves/mm), conjugated with the camera via a 1:1 imaging system, generates the reference wave required for off-axis holography, as its first diffraction order. Adequate beam blocks placed into the Fourier plane of the grating eliminate all other diffraction orders.

The setup outlined above ensures broadband off-axis interference between the signal and reference arms by meeting two key requirements. First, wavefront matching is ensured by performing angle scanning and off-axis holography using a diffraction grating, which allows interfering temporally short waves on large 2D detectors^36,37^ (Figure 2b). Second, interference over the entire spectral bandwidth is enabled by carefully matching the spectral phases, or chirps, of signal and reference beams at the camera (Figure 2c), which is possible via spectral interferometry^38^ (Supporting Information 1). Importantly, in this proof-of-concept work, we control the relative spectral phase difference between the two fields and not the absolute spectral phase of the fields, which would be required for ultrafast 3D transient absorption microscopy. This reduces the experimental complexity compared to operating with transform-limited pulses.

In a typical experiment, we record 60 off-axis holograms while systematically changing the illumination k-vector via grating rotation in steps of 6°. The laser repetition rate was 15.59 MHz, and the camera integration time was 1 ms to ensure that potential low-frequency vibrations do not degrade the interference contrast. In all experiments, we recorded sample and background information by capturing image stacks of objects of interest alongside identical stacks of empty regions, respectively.

### 2.2 Spectral Resolution

**Figure 3:**
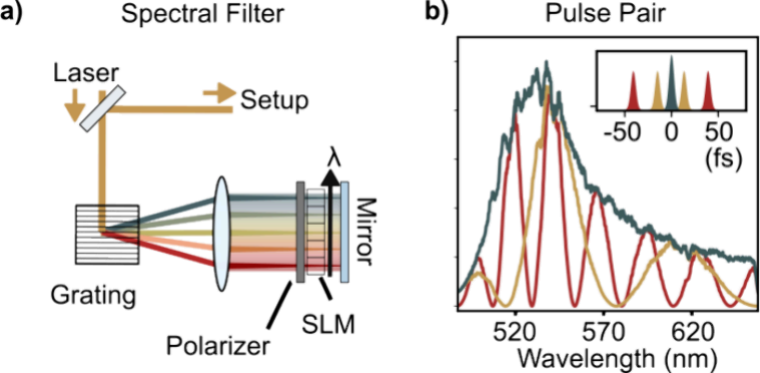
Spectral imaging configuration. (a) A 4f-grating zero-dispersion pulse shaper equipped with a polarizer and spatial light modulator (SLM) in the back-focal plane manipulates the pulse spectrum. (b) Selected spectra used for time-domain Fourier transform, or pulse-pair, imaging at a nominal spectral resolution of 6.25 THz, or 6.9 nm, at 575 nm. The inset shows a schematic sketch of the intensity of the DC pulse, together with the time-delayed satellite pulse pairs.

The imaging system described in Figure 2 enables broadband ODT but lacks spectral resolution. We provide this much-needed capability via pulse-shaping to perform Fourier-transform spectroscopy,^1^ using a simple Fourier filter, in combination with a spatial light modulator (SLM), placed between the supercontinuum laser and the ODT setup (Figure 3a). Our implementation is based on the established mirrored 4f-design in which a grating disperses the incoming light, which is then Fourier transformed by a lens onto the liquid-crystal mask of the SLM (*Jenoptik SLM- S640d*) followed by back-reflection through the system. A polarizer placed in front of the SLM allows phase and amplitude control in a straightforward manner.

Time-domain Fourier transform measurements^1^ via a so-called “pulse pair” (Figure 3b) are a sampling strategy that is especially popular in ultrafast spectroscopy as it favorably integrates with the available broadband light sources.^39^ In brief, the SLM modulates the frequencies, ν, of the dispersed spectrum according to *M*(ν, τ) = | cos ((ν – ν_0_) * π*τ)|^2^, with ν_0_ being the carrier frequency that generates a pulse pair with effective time delay τ. Operating in a rotating frame^40,41^ allows Nyquist-sampling the τ = 0–40 fs temporal range in 24 steps. A pixelwise one-dimensional fast Fourier transform (FFT) along the SLM-generated time-delay axis retrieves the spectral components of interest.

### 2.3 Reconstruction Procedure

**Figure 4:**
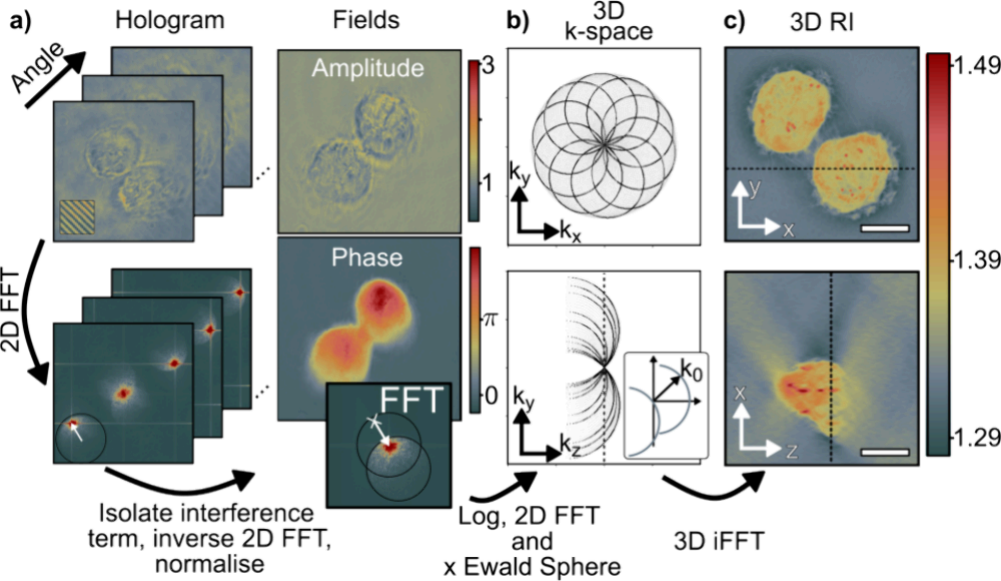
From raw holograms to 3D tomograms. (a) A set of holograms (with and without sample) is recorded at different illumination angles and then Fourier-processed to retrieve normalized amplitude and phase images. The top inset shows the interference fringes of the hologram. The bottom inset corresponds to the 2D FFT of the normalized image. The white arrows show the (lateral) illumination beam in the k-space and the shift due to normalization. (b) The object’s scattering potential in 3D k-space is sampled using the 2D FFT of the normalized fields and the corresponding Ewald sphere of each illumination angle. Only a few subsets are highlighted for clarity. The inset shows the shift of the Ewald sphere for an illumination with k-vector . (c) An inverse 3D FFT of the Ewald sphere retrieves the 3D, real space, RI of the object. Dotted lines represent the respective image slices. Scale bar: 10 μm.

The setup outlined above yields data sets containing spectrally resolved sample holograms, acquired at each illumination angle, alongside a second background data set, acquired in an empty region. These data allow reconstruction of the spectrally resolved 3D RI of the sample. Out of the many different reconstruction strategies^19,42−45^ available, we chose the Rytov approximation,^16,17,20,46^ a widely used approach that enables straightforward 3D reconstructions of the real part of the RI based on a set of complex 2D recordings. The reconstruction workflow, from raw holograms to 3D volumetric images, is schematically outlined in Figure 4 and explained in detail in the Supporting Information 2. Before performing the actual reconstruction, we extract the complex interference terms from the image stacks, following established Fourier-filtering based^47^ hologram-processing routines (Figure 4a). We retrieve the complex field, defined as *f*_0_(*x*, *y*, τ, ϑ), with *x* and *y* being the spatial coordinates, ϑ the illumination angle, and τ the delay between the satellite pulses. We apply a one-dimensional pixelwise FFT to extract the wavelength-dependent field from the temporal interferogram to yield complex fields *f*_0_(*x*, *y*, λ, and ϑ) as a function of wavelength and angle (Supporting Information 3). The same procedure is applied to the background holograms yielding *f*_*bg*_(*x*, *y*, λ, ϑ), which allows eliminating amplitude and phase contributions that do not originate from the sample, such as nonuniform illumination profiles or residual, static, phase differences between signal and reference waves. The background fields are, further, used to retrieve the illumination *k*-vectors, , for each angle and wavelength (Supporting Information 4).

Following amplitude and phase image retrieval, we move on to reconstructing the 3D volumes, processing each wavelength separately, following refs (17,20). In simple terms, illuminating the sample at different angles yields projections that encode 3D information into a complex 2D image stack. Knowledge of the illumination angle allows placing this 2D information into a computationally generated 3D k-space on a so-called Ewald sphere. Once the entire information, for all angles but at a fixed wavelength, has been inserted, an inverse FFT yields the 3D real space image, or RI, for one wavelength component. Figure 4 summarizes the entire workflow from raw holograms of fixed HeLa cells to xy- and xz-cuts of the tomogram. Supporting Information 5 provides a short discussion of the artifacts in ODT due to the missing cone problem and the choice of the Rytov approximation.

## 3 Results and Applications

Our method retrieves spectrally resolved tomograms via Fourier transform spectroscopy but might suffer from potential experimental artifacts due to, for example, loss of interference as a result of residual spectral phase or spatial misalignment. The as-recorded broadband holograms exhibit high fringe contrasts of around 70% confirming high-quality broadband interference (Supporting Information 6). To further validate the approach, we compared the electric fields, as retrieved from the broadband Fourier-transform holographic measurements, to an established, narrowband, slit-scan approach (Supporting Information 7).^28−30^ The slit scan reconstructs broadband images from many narrow-band observations at varying wavelengths. As such, it conveniently eliminates potential sources of artifacts but is, from a temporal-resolution perspective, incompatible with ultrafast observations. Irrespective, the two modalities deliver comparable spectral observations even though the temporal coherence lengths, , of individual observations differ by more than an order of magnitude: an ideal handle to ensure artifact-free observations.

**Figure 5:**
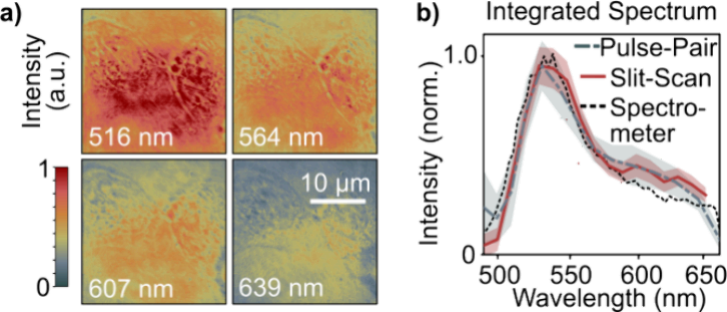
Proof of broadband interference. (a) Intensity (*A*_s_*A*_r_) images underlying the HeLa cell sample reported in Figure 6 shown for one illumination angle at representative wavelengths obtained via pulse-pair imaging. (b) Normalized integrated intensity images over 60 angles obtained via pulse pair imaging (dashed line), via a sequence of narrowband images (red line), and normalized source spectrum as measured by a spectrometer (dotted line). The shaded areas correspond to two standard deviations.

Figure 5a shows raw intensity images obtained for one illumination angle, as extracted from holograms recorded on HeLa cells. The unnormalized intensity images directly report on the intensity of the interference term, *A*_s_*A*_r_, with *A*_s_ and *A*_r_ being the signal and reference amplitudes, respectively. The images show a strong wavelength dependence, which should, in principle, reflect the spectrum of the supercontinuum source in the case of perfect interference over the entire spectrum.

To benchmark the quality of our data, we spectrally integrate the intensity images and then combine all illumination angles into one data point for each wavelength for both the Fourier transform approach and the slit scan. Further, we acquire ground-truth spectra using a commercial spectrometer (*Ocean Optics*). Figure 5b compares the normalized spectral intensities obtained by using the three approaches. Overall, we observe near-perfect agreement between the slit scan and pulse pair methods. Minor discrepancies with respect to the spectrometer ground-truth data toward the red edge of the spectrum are apparent, which we attribute to potentially nonperfect quantum- or grating-efficiency calibrations of either the imaging camera or the spectrometer as well as marginal differences between beamsplitter reflectivity and transmissivity. Overall, the data suggest artifact-free ultrabroadband tomographic observations.

**Figure 6:**
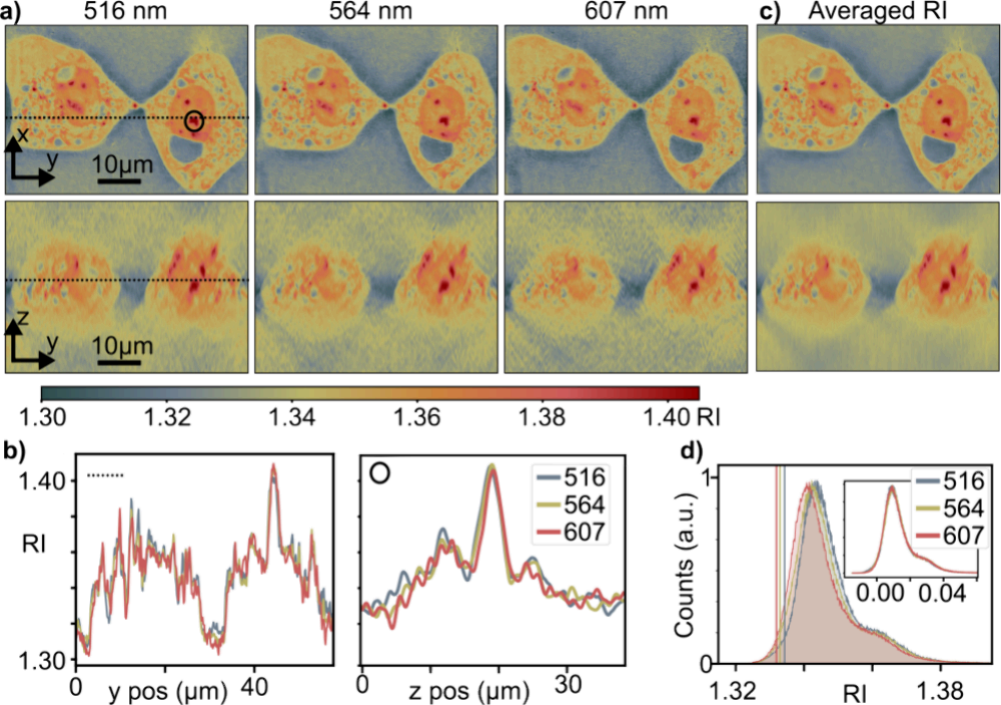
Wavelength resolved 3D tomography of HeLa cells. (a) *xy*- and *xz*-projections of Hela cells imaged with the pulse-pair method and reconstructed for different wavelengths. The lines correspond to the respective cut in the *xy*- and *yz*-images. (b) RI along *y* and *z* (solid line and red circle in panel (a), respectively). (c) Averaged RI obtained using nine reconstructions in the wavelength range of 516 to 607 nm. (d) RI distribution for the HeLa cell located on the right side of the images shown in panel (a), the vertical lines indicate the RI of the surrounding medium (water). The inset shows the histograms with the corresponding surrounding medium subtracted.

An important application of ODT is imaging biological samples such as mammalian cells. To this end, Figure 6 presents spectrally resolved broadband tomography experiments, performed on HeLa cells and acquired with the pulse pair approach. Figure 6a shows *xy*- and *yz*-tomogram projections obtained for two fixed HeLa cells at three Fourier-extracted wavelengths of 516, 564, and 607 nm. We observe the typical morphology for adherent cells alongside the expected RI differences between the nucleus and cytoplasm. The small regions of considerably higher RI might be endosomes, lipid droplets, or otherwise aggregated biological material. The seemingly void regions, with RI-values comparable to water, are most likely due to fixation-induced disruption of the structural integrity of the cells, a problem of paraformaldehyde fixation that is especially visible with holographic and tomographic observations.^48^ Overall, pulse-pair-based ODT yields broadband, spectrally resolved, tomograms whose quality is comparable to those acquired via state-of-the-art narrowband approaches.^49^ Spectrally, we only observe minor differences between the images, with a slight noise increase toward the red part of the spectrum. Figure 6b confirms this notion by comparing RI measurements for the three wavelengths along representative x- and z-cuts. These observations are consistent with the fact that the RI of biological materials changes slowly in the visible spectral range. Further, it can be observed that the RI drops below the RI of water at the edges of the cells, which is due to the well-known missing cone artifact (Supporting Information 5). The overall agreement of the RI between all wavelengths allows generating wavelength-averaged tomograms as the RI mean. Figure 6c shows an averaged RI distribution obtained for nine distinct wavelengths in the 516–607 nm observation window. Importantly, such averaging is only possible once the 3D real space RI distributions are obtained due to the wavelength-dependent nature of the k-vectors.

To gain more quantitative insight, we compare the RI distributions of the entire right HeLa cell for different wavelengths with those of water (Figure 6d). We observe RI changes as a function of wavelength with a slight decrease for increasing wavelengths. Importantly, the histograms show shifts similar to water, as expected given that off-resonant organic matter and water exhibit comparable chromatic dispersion in our spectral range. This observation suggests that the Rytov approximation, albeit underestimating the RI values, is spectrally accurate. The minor differences in the histograms, after normalizing them to their respective water RI (inset Figure 6d), are most likely due to the wavelength dependent spatial resolution which slightly reduces the apparent RI for small objects contained in the lower RI surrounding water (Supporting Information 8).

**Figure 7:**
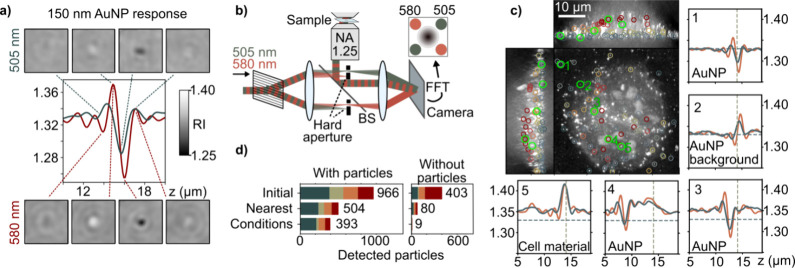
Multiplexed two-color tomography for robust 3D particle identification. (a) Rytov-extracted RI-signals for 150 nm Au NPs at 505 nm (blue) and 580 nm (red). (b) Two reference-waves enable multiplexed hologram acquisition at 505 and 580 nm. BS: beamsplitter. (c) Maximum RI projection values along *x*, *y*, and *z* (grayscale) alongside detected NPs (green). The color scale indicates the *z*-position. Line plots show the RI along *z* for selected positions. The horizontal line indicates the RI of water, and the vertical line indicates the *z*-position of the cover glass. (d) Statistics of four respective measurements with and without Au NPs using increasingly more rigorous filtering. Each color represents one measurement.

Figure 6 highlights the ability of ultrabroadband ODT to deliver high-quality, spectrally accurate, 3D reconstructions. To take the first steps toward interrogating spectrally resonant systems, we incubate HeLa cells with Au nanoparticles (NPs). The representative 3D response of an isolated single 150 nm Au NP at 505 and 580 nm (Figure 7a) suggests that spectrally resolved ODT should be well-suited for identifying such particles in scattering biological matter. To test this hypothesis, we employ a tomographic implementation of a multiplexed holographic detection scheme, based on our previous design.^50^ Our approach simultaneously records tomograms at 505 and 580 nm in order to identify Au NPs within cells. Figure 7b summarizes the necessary experimental modifications to implement two color-detection. Prior to the imaging system, we select the two colors, 505 and 580 nm, from the broadband light source using a hard-aperture Fourier filter in front of the SLM (Figure 3). Multiplexed two-color interference is ensured by selectively blocking the respective colors after the first lens following the 2D grating in the reference path. Blocking one of them in each reference beam, respectively, retrieves two references with different colors and *k*-vectors. The (complex) images of the two colors are then retrieved by Fourier processing of the spectrally multiplexed hologram, as discussed in detail previously.^50^

Figure 7c summarizes a typical data set obtained on a fixated Au-incubated HeLa cell where we highlighted a few “nanoparticle-like” and “cell-like” nano-objects. Comparing typical observations with the single-particle measurements (Figure 7a), suggests that the distinct Au NP RI-responses allow two-color-based identification.

To gain systematic insight, we first identify potential Au NPs over the entire 3D volumes by calculating so-called Haar features used in digital image processing.^51^ The Haar feature consists of subtracting the summed RI over a certain number of pixels (line, area, or volume) from the sum over an adjacent region. This operation identifies the strong RI change around the position of Au NPs (Figure 7a). We perform this operation along the *z*-dimension. More specifically, at 505 nm, we subtract the sum over 11 pixels from the adjacent 11 pixels, and at 580 nm, we introduce an additional 3 pixel gap between the regions. These values were selected empirically to yield the highest contrast. Thresholding at 0.008 (505 nm) and 0.01 (580 nm) RI contrast yields a list of possible particles for both wavelengths. On average, for the illumination wavelength of 580 nm, around 250 particles are detected for an Au NP-incubated cell and 100 particles if no Au NPs are present. Using four samples each, we detected 966 particles (Au incubated) and 403 particles (not incubated). The number of initially detected particles at 505 nm is about twice as high, as the contrast of the particle response is weaker and thus more comparable with the background (data not shown).

To reduce the number of false positives, we make use of the two-color detection. First, we eliminate all particles that are not present in both detection channels and only keep those that are present in both with a maximum lateral distance of 240 nm and axial distance of 660 nm. This step removes around 80% of the false positives in the control group without Au NPs (Figure 7d, “Nearest”). To further reduce this number, we introduce additional conditions based on the typical RI responses (Figure 7a or Supporting Information 9). These are that the RI at 580 nm is smaller than at 505 nm and that the contrast of the Haar feature at 580 nm is higher than at 505 nm. Further, the *z*-position of the minimum RI at 580 nm is smaller than at 505 nm, a feature also apparent in Figure 7a and possibly originating from the different focus of the objective lens.^23^ Also, we set an absolute threshold to both features of 0.05 to remove clear outliers. Lastly, we remove particles that were present in the background image by exploiting the fact that the Haar Features are reversed along z for these in comparison with the original particles. Using the two-color approach, we obtain 2.25 particles per nonincubated sample and 393 particles in the incubated sample. Reassuringly, a similar number of particles is eliminated from both samples, which suggests that our constraints selectively remove non-Au particles (Figure 7d).

## 4 Summary and Conclusions

To summarize, we experimentally implemented high-resolution, spectrally resolved, broadband ODT over bandwidths that are compatible with high temporal resolution experiments. We successfully recovered signals covering essentially the entire visible spectral range (500–650 nm), as validated by comparing to established narrow-band spectral scanning alternatives. These capabilities directly enable highly temporally resolved experiments, which have, thus far, been incompatible with ODT implementations. Further, we showed that multicolor tomography allows robust NP identification, based on known RI differences between biological and nonbiological matter. Taken together, our results provide the necessary experimental framework for time-resolved 3D imaging of photoinduced RI changes in both synthetic and biological matter.

Moving forward, we identify two areas that require further improvement. Experimentally, the illuminating angles were created by a rotating grating. While being experimentally advantageous the physical rotation is slow compared to typical camera frame rates. Detecting 24 color-multiplexed holograms at 60 angles required 18 min, including holograms for a background. The theoretically necessary acquisition time is <3 s thus leaving plenty of room for improvement. Second, rotating the grating generates a cone-shaped illumination geometry. By using diffractive optics, such as spatial light modulators or digital micromirror devices,^25,52^ the imaging speed could be greatly improved. Assuming typical modulation frequencies and the same parameters outlined above, such a strategy should allow full tomogram acquisition in <6 s. Furthermore, diffractive optics would allow to vary not only the azimuth but also the polar illumination angle, thus further boosting the axial resolution.^18^ Computationally, the Rytov approximation provided a good framework in this work (Supporting Information 5). However, the implementations of algorithms (i) retrieving the real and imaginary part of the RI^19,21^ and (ii) mitigating artifacts of the missing cone problem to increase the fidelity of the 3D volume^17,43−45^ and be applicable to scattering media^53^ to resolve thick tissue are desirable. Given the current move toward biomedical imaging in the deep ultraviolet spectral range,^54^ we expect that algorithms for 3D RI-retrieval of highly scattering media as well as thick tissue will become available in the near future.

The methodology developed here bridges the gap between ODT and broadband ultrashort pulses and is directly applicable to pump–probe ODT with the only modifications necessary being to exchange the temporally chirped supercontinuum source employed here with a transform-limited temporally compressed source and to add an excitation pulse with controllable time delay. Our work is directly compatible with chemically sensitive microscopy, such as stimulated Raman scattering or photothermal imaging, where it provides a straightforward path to using broadband light sources combined with widefield detection and phase-sensitive, heterodyne, detection. We expect that the ultrafast phototransient response will give a high contrast for nano particle detection^13^ in otherwise highly scattering media and will permit to study complex time-resolved processes in three dimensions.

## Supporting Information Available

Additional experimental details and methods alongside a discussion of the computational steps used for retrieving the 3D spectrally resolved images (PDF)

## Notes

A preprint version of this article has been submitted to the arXiv: Hörmann, M.; Camargo, F.V.A.; van Hulst, N.F.; Cerullo, G. & Liebel M. Ultrabroadband Optical Diffraction Tomography. 2024, 2401.07391. arXiv. https://arxiv.org/abs/2401.07391 (accessed August 19, 2024).
