# Supplementary material for: Ultrabroadband Optical Diffraction Tomography
Source: ACS Photonics. 2024 Aug 27;11(9):3680–7. doi: 10.1021/acsphotonics.4c00797 (PMC11413850; doi:10.1021/acsphotonics.4c00797)
Supplement: Supplementary file 1 — ph4c00797_si_001.pdf [file ph4c00797_si_001.pdf]

Supplementary Information

Ultra-broadband Optical Diffraction Tomography

Martin Hörmann[1], Franco V. A. Camargo[2], Niek F. van Hulst[3,4], Giulio Cerullo[1,2], and Matz Liebel[3,5*]

[1]*Dipartimento di Fisica, Politecnico di Milano, Piazza L. da Vinci 32, 20133 Milano, Italy*

[2]*Istituto di Fotonica e Nanotecnologie-CNR, Piazza L. da Vinci 32, 20133 Milano, Italy*

[3]*ICFO – Institut de Ciencies Fotoniques, The Barcelona Institute of Science and Technology, Av. Carl Friedrich Gauss, 3, 08860 Castelldefels, Barcelona, Spain*

[4]*ICREA – Institució Catalana de Recerca i Estudis Avançats, Passeig Lluís Companys 23, 08010 Barcelona*

[5]*Department of Physics and Astronomy, Vrije Universiteit Amsterdam, De Boelelaan 1081, Amsterdam, 1081 HV, The Netherlands*

**email: m.liebel@vu.nl*

Contents

1. Matching Spectral Phase of Reference and Signal Pulses 2
2. Reconstruction Procedure for Rytov and Born Approximation 2
3. Extracting Spectra from Pulse-Pair recorded Holograms 4
4. Retrieving k_{0x}, k_{0y} and k_{0z} 5
5. Missing Cone Problem in ODT 5
6. Interference Contrast in Holograms 6
7. Illumination via Wavelength-Scanning 6
8. Wavelength Dependent k-Space and Resolution 7
9. Spectral Response of 150 nm Au Nanoparticles 7

1. Matching Spectral Phase of Reference and Signal Pulses

To achieve interference over the entire wavelength range of interest it is necessary to carefully balance the spectral chirp of the "Reference" and "Signal" arms of the broadband optical diffraction tomography (ODT) setup (Figure 2). We mimic the highly dispersive elements present in the microscope by adding dispersive components to the reference path, mainly in the form of achromatic lenses, to approximate the highly dispersive materials used in high numerical aperture microscope objectives. A pair of wedge prisms fine-adjusts the chirp.

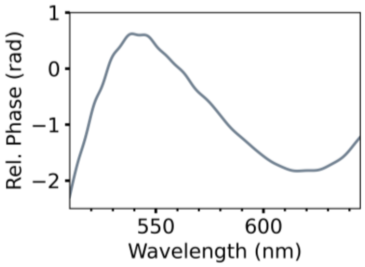

Figure SI 1: Relative phase (in radians) after balancing the dispersion in the reference arm of the ODT setup.

To quantify the chirp difference between the two interferometer arms, we rely on spectral interferometry by placing a commercial spectrometer (*Ocean Optics*) at the position of the camera. As such, we directly record spectrally resolved interference between reference and signal waves. Fourier analysis[1,2] directly retrieves the difference between the spectral phases of the pulses, which corresponds to a difference between the arrival times of their different frequency components. We add dispersive elements (lenses, windows) to the reference path until the relative spectral phase becomes as flat as possible.

Figure SI 1 shows the obtained wavelength dependent relative spectral phase where the residual higher order dispersion is, most likely, due to material differences where especially high numerical aperture objectives often contain highly dispersive, specialized, optical glass.

2. Reconstruction Procedure for Rytov and Born Approximation

The following reconstruction is carried out for each wavelength separately. We start with the complex extracted fields $f_0(x, y, \vartheta)$ and $f_{bg}(x, y, \vartheta)$. For the Rytov approximation, we normalize the as-retrieved fields [3]:

$$f_{norm,\vartheta}(x, y) = \frac{f_0(x,y,\vartheta)}{f_{bg}(x,y,\vartheta)} = A_\vartheta(x, y) \exp[\mathrm{i}\, \varphi_\vartheta(x, y)],$$

and then apply the complex logarithm:

$$f_{Ryatov,\vartheta}(x, y) = \log\left(f_{norm,\vartheta}(x, y)\right) = \mathrm{i}\, \varphi_\vartheta(x, y) + \log(A_\vartheta(x, y)) \,.$$

For the Born approximation we use[3]:

$$f_{norm,\vartheta}(x, y) = \frac{f_0(x, y, \vartheta) - f_{bg}(x, y, \vartheta)}{f_{bg}(x, y, \vartheta)} \,.$$

We omitted the illumination angle, ϑ, to simplify the discussion but note that the operation has to be performed for all fields. Then the process is for both approximations the same. A 2D FFT yields the k-space distribution, $\bar{f}_{Ryatov}(k_x, k_y)$, with the illumination k being shifted to the center of the k-space, or DC, due to the background normalization (inset Figure SI 2a). The next step is to use knowledge of the spatial frequencies k_x, k_y as well as the illumination spatial frequencies k_{x0}, k_{y0} to reconstruct the Ewald sphere. We require knowledge of $k_{Ewald}(k_x, k_y)$, the k_z-component on the Ewald sphere, which is accessible via the refractive index of the surrounding medium, n_m, and the wavelength λ, $k_0 = \frac{2\pi}{\lambda}$:

$$k_{Ewald}(k_x, k_y) = \sqrt{(n_m k_0)^2 - (k_x + k_{x0})^2 - (k_y + k_{y0})^2}$$

if $(k_x + k_{x0})^2 + (k_y + k_{y0})^2 < (k_0 * n_m)^2$, otherwise zero,

where we re-introduced the original illumination information k_{x0} and k_{y0} to move the Ewald sphere on top of the circle in k-space (inset Figure SI 2a). Once $k_{Ewald}(k_x, k_y)$ is determined, we scale the Rytov-retrieved k-space distributions as:

$$\bar{f}_{Ewald}(k_x, k_y) = \frac{\mathrm{i}}{\pi}\, k_{Ewald}(k_x, k_y)\ \bar{f}_{Ryatov}(k_x, k_y)\,.$$

Finally, each of the values is assigned to its position within a 3D scattering potential $\bar{F}(k_x, k_y, k_z)$ of the object of interest, which lies on the shifted semi-sphere: the Ewald sphere. The k_z position is inferred as follows:

$$k_z(k_x, k_y) = k_{Ewald}(k_x, k_y) - k_{z0}\,.$$

This process is repeated for all angles ϑ to fill the 3D potential, as shown in Figure SI 2b. For points which are covered by multiple acquisitions, using different acquisition angles, mean values are calculated. Finally, an inverse 3D FFT retrieves the distribution in real space, which is converted from scattering potential to the refractive index (Figure SI 2c):

$$RI(x, y, z) = n_m \sqrt{1 - F(x, y, z) * \left(\frac{\lambda}{2 * \pi n_m}\right)^2}\,.$$

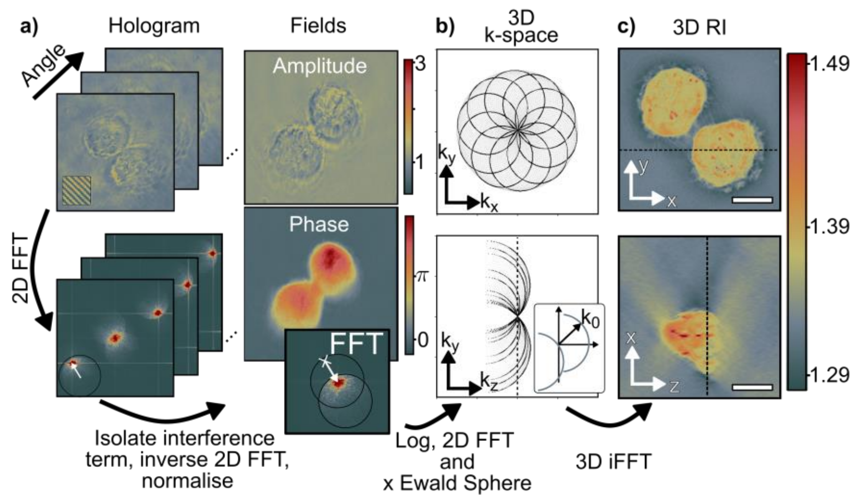

Figure SI 2, From raw holograms to 3D tomograms. a) A set of holograms (with and without sample) is recorded at different illumination angles and then Fourier-processed to retrieve normalized amplitude and phase images. The top inset shows the interference fringes of the hologram. The bottom inset corresponds to the 2D FFT of the normalized image. The white arrows show the (lateral) illumination beam in k-space and the shift due to normalization. b) The object's scattering potential in 3D k-space is sampled using the 2D FFT of the normalized fields and the corresponding Ewald sphere of each illumination angle. Only a few subsets are highlighted for clarity. The inset shows the shift of the Ewald sphere for an illumination with k-vector $\underline{k}_0$. c) An inverse 3D FFT of the Ewald sphere retrieves the 3D, real space, RI of the object. Dotted lines represent the respective image slices. Scale bar: 10 µm.

3. Extracting Spectra from Pulse-Pair recorded Holograms

As described in the main text in Section 2.2 we record holograms for which the spectrum is modulated by a cosine square to enable Fourier transform based spectrally resolved imaging. For each modulation frequency ν we record one hologram.

We then retrieve the spectrum on a pixel-by-pixel basis. We define H(x, y, n_ϑ) as the value of the pixel at position x and y and n_ϑ as the n-th acquisition for the modulation ϑ. Then we retrieve one-dimensional arrays over all n_ϑ acquisitions which we call f_{xy}.

Our original acquisition has 24 modulation frequencies, covering a 0- 40 fs temporal delay range. Before performing the fast Fourier transformation (FFT) over f_{xy}, we invert f_{xy}, delete it's first component and append it to the original f_{xy}, as shown in Figure SI 3a. Following this operation, we perform the one-dimensional FFT over the appended array of f_{xy}, yielding the spectral information of interest (Figure SI 3b). The wavelength region of interest is highlighted.

Keeping in mind that we use a carrier frequency with wavelength $\lambda_{carrier}$, we retrieve its frequency as $\nu_{carrier} = \frac{c}{\lambda_{carrier}}$. We calculate the spectral modulation corresponding to a time delay, τ, as ϑ: $M(\nu, \tau) = |\cos\big((\nu - \nu_0)\,\pi\,\tau\big)|^2$.

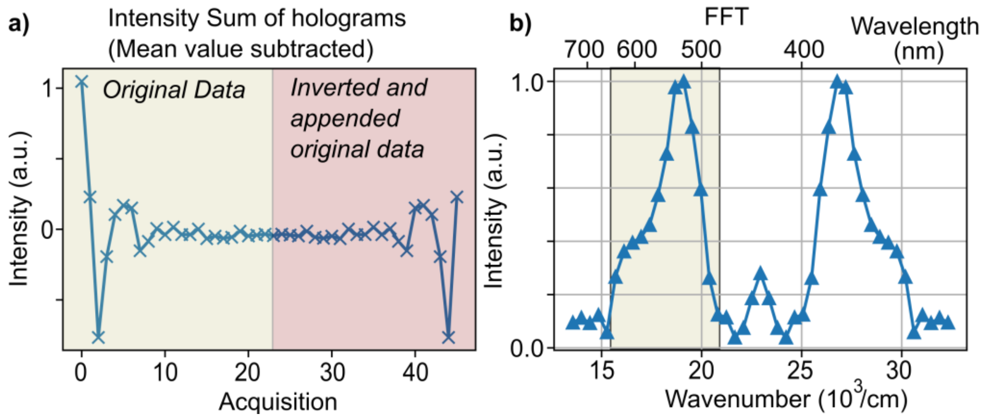

Figure SI 3: Pixelwise FFT extraction for pulse pair spectroscopy. a) Prior to the fast Fourier transformation (FFT) the one-dimensional array (orange) is inverted and appended to the back of the original array (blue). As an example we plotted here the absolute value, whereas the fields are complex. b) Resulting FFT, the spectral region of interest is highlighted in orange.

4. Retrieving k_{0x}, k_{0y} and k_{0z}

Tomographic reconstruction requires precise knowledge of the illumination angle, or k-vectors. We use the background fields, e.g. images recorded in an empty region of the sample, to retrieve the original illumination vectors. We measure the phase of each illumination angle (two-dimensional array) and unwrap it to retrieve a phase which allows calculating the k-vectors as $k_{x0/y0} = \frac{\Delta\varphi_{x/y}}{p}$, with $\Delta\varphi_x$ and $\Delta\varphi_y$ being the phase change per pixel and p the physical pixel size at the sample plane. The k_z-component is retrieved via $k_{z0} = \sqrt{(k_0 * n_m)^2 - k_{x0}^2 - k_{y0}^2}$, with $k_0 = \frac{2\pi}{\lambda}$, λ the wavelength of the incoming light and n_m the refractive index of the surrounding medium. Figure SI 4 shows the retrieved k-vectors and angles of a pulse-pair scan for all wavelengths.

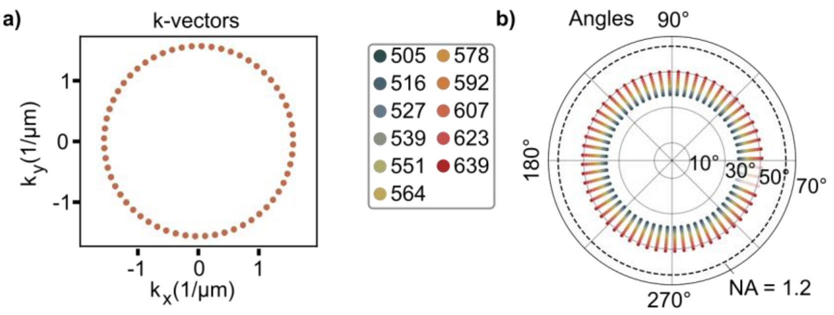

Figure SI 4: k-vector retrieval. a) k-vectors retrieved for all wavelengths used in FFT approach (505 nm to 639 nm). b) Illumination angles extracted for all wavelengths.

5. Missing Cone Problem in ODT

The limited illumination angles allow to fill the k-space of the object only partially, as apparent in Figure 4b. This is known as missing cone problem. The consequences can be seen in the reconstruction in Panel 4c as a slight blurring along the z-coordinate and RI values below water at the edges of the object.

Furthermore, the overall RI value is slightly underestimated. To this end, many iterative solvers exist trying to circumvent this problem[3–5], or to make ODT applicable outside the Rytov approximation for scattering media[6]. These algorithms mitigate the missing cone problem and increase the fidelity of the retrieved 3D objects. However, they do not gain further real information, e.g. record information beyond the diffraction limit. Thus, in the context of this article, which mainly focuses on implementing ODT with broadband pulses, we stick to the Rytov approximation as it (i) best visualizes the recorded experimental data (only a two-dimensional phase unwrapping algorithm is necessary) without the use of complex iterative solvers, which could introduce their own artefacts, and (ii) is sufficiently accurate for the data presented here (ignoring the artefacts due to the missing cone problem).

6. Interference Contrast in Holograms

Figure SI 5a shows a recorded hologram at a single illumination angle in which the entire unmodulated broadband spectrum was utilized. The inset in Figure SI 5a reveals high fringe contrast. To better visualize the contrast, we plot the indicated line-cut through the image (Figures SI 5a,b). The estimated fringe contrast is high and uniform over the entire detector thus suggesting successful broadband-interference. We attribute the deviation from unity contrast to non-perfectly matched signal and references waves as well as back-reflected light generated by the uncoated Ronchi gratings used in this work.

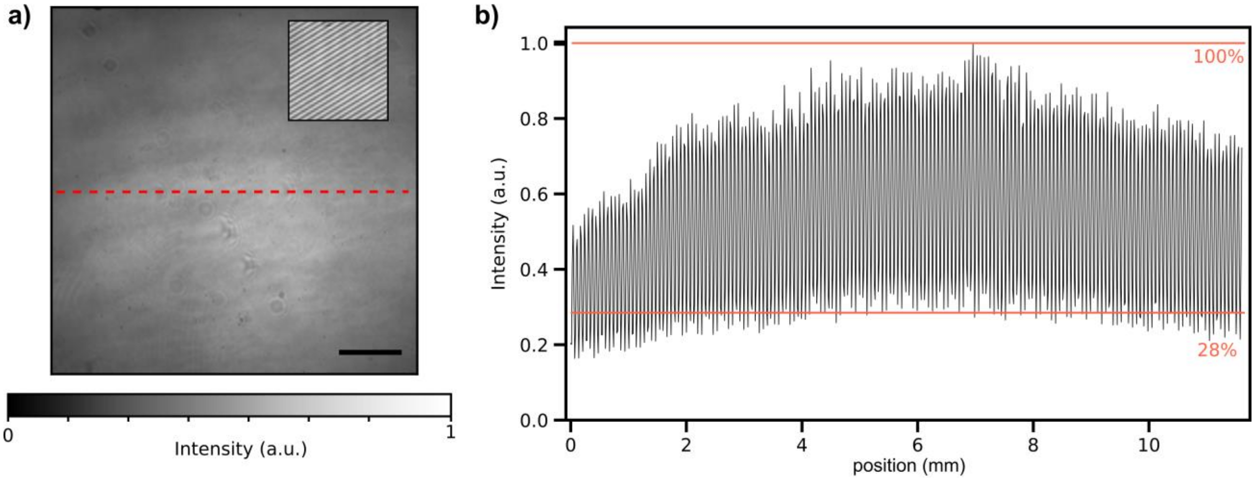

Figure SI 5: Holographic interference contrast. a) Hologram using the entire spectrum at a single illumination angle. Inset: Magnified representative area indicating interference fringes. b) Normalized line-cut through the image as indicated in a). Ther red lines show approximate maxima and minima.

7. Illumination via Wavelength-Scanning

In the context of the proof-of-principle experiments presented in Figure 3 of the main text we implement a wavelength scanning approach. The spatial light modulator sweeps illuminations with 10 nm bandwidth. Figure SI 6 shows the spectra after the Fourier filter recorded with a commercial spectrometer (*Ocean Optics*). In order to remove the leakage of the SLM, one acquisition with all wavelengths blocked is acquired and in a post-processing step subtracted from the other acquisitions.

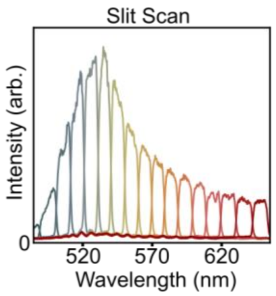

Figure SI 6: Recorded spectra used for wavelength-scanning.

8. Wavelength Dependent k-Space and Resolution

ODT allows extending the spatial resolution. Following reference[7], the resolution is extended up-to two fold $k_x = k_0 * n_m * \sin(\theta)$, depending on the illumination angle θ between axial and lateral illumination k-vectors. Thus, the resulting lateral resolution is:

$$d_{lat} = \frac{1}{2\, k_{x,NA} + 2\, k_x}$$

with $k_{x,NA} = k_0\, n_m \sin\,(\theta_{NA})$), θ_{NA} is maximum angle defined by NA ($NA = \sin(\theta_{NA})\, n_m$).

If the illumination is equivalent to θ_{NA}, then $d_{lat} = \frac{\lambda}{4NA}$ which is the maximum resolution limit. Analogously, for an illumination with cone-geometry , the axial resolution reads as

$$d_{ax} = \frac{1}{n_m\, k_0\, (1 - \cos(\theta_{NA}))}\,.$$

Figure SI 7 shows the wavelength dependent, calculated, spatial resolutions.

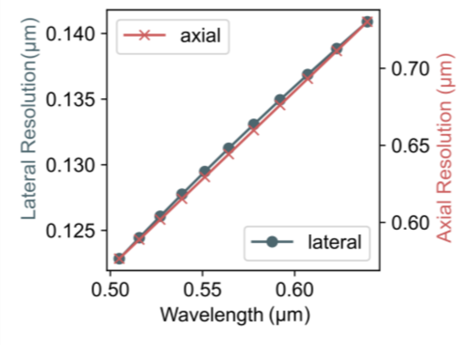

Figure SI 7: Theoretical lateral and axial resolution in ODT with cone-illumination calculated for the experimental parameters and illumination angles from Figure SI 4.

9. Spectral Response of 150 nm Au Nanoparticles

To further validate the setup, we recorded broadband tomographic images of gold nano particles (Au NPs) with a diameter of 150 nm immobilized on glass. In contrast to the off-resonant HeLa cells, Au NPs show

a strongly wavelength dependent RI in the visible spectral range: the RI of bulk gold strongly decreases with wavelength[8]. Figure SI 8a shows an xy-plane cut in the vicinity of a cover glass surface of a reconstructed Au NP tomogram obtained with a pulse pair. We observe multiple Au NPs, immersed in water and immobilized onto a glass coverslip, that show a RI that is lower than water, as expected based on the RI of bulk gold. As previously, we examine the wavelength dependence of the RI (Figure SI 8b) where we note a marked decrease with increasing wavelength when analyzed in the respective tomogram plane. However, the xz-projections through the center of the representative Au NP show a RI increase followed by a decrease, especially at longer wavelengths. To rationalize this observation, we concentrate on averaged RI line cuts, obtained by combining the data of all Au NPs (Figure SI 8c). The RI at 607 nm shows a derivative profile with a decrease to a minimum of around 1.26 as well as an increase to up to 1.39. Based on the bulk RI of gold, one might have expected RI values of around 0.5-0.8. Given that the particles employed are strongly scattering, reconstruction-artefacts due to loss of information are a likely cause for this discrepancy. A further reason for the only partial recovery of the RI values is the small size of the Au NPs compared to the 130 / 650-nm lateral/axial point spread function of the system, which essentially averages the RI response of the NPs with that of the surrounding medium.

To ensure that no bandwidth-associated artefacts are present, we first compare the particles' responses as measured via a slit scan with the pulse-pair methods. Figure SI 8c,d highlight that both methods yield near-identical results. In other words, the RI-oscillations are not due to an experimental error. To gauge the impact of the reconstruction procedure, we compare the RI values as obtained via the Rytov approximation (Figure SI 8d) to the one obtained following a prominent alternative: the Born approximation[3] (Figure SI 8e). The latter is considered valid for sample-induced phase changes below $\frac{\pi}{2}$, [3] a condition that is fulfilled by the Au NPs. Indeed, the positive contribution vanishes with the Born approximation, which suggests that it is a more accurate reconstruction procedure for the Au NPs. From a computational perspective, more advanced algorithms are likely to yield even better results [4,5,9,10]. Another reason for spectral differences could be the k-space filling, which is wavelength dependent.

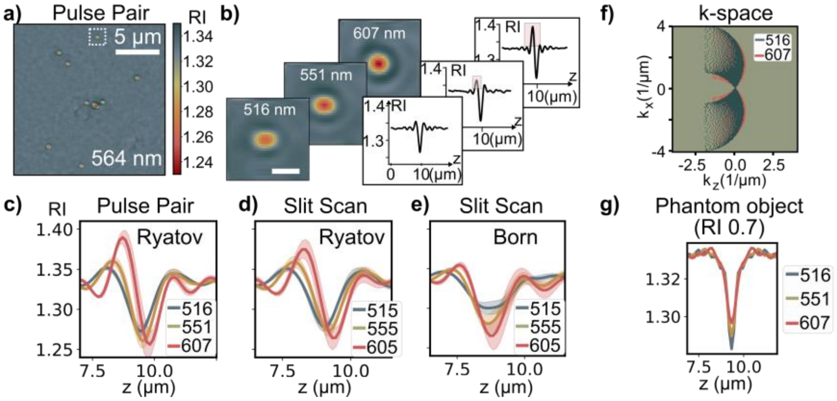

Figure SI 8, Wavelength resolved 3D-reconstructions of 150 nm Au NPs using different approaches. a) Image of Au NPs on top of a glass substrate and immersed in water obtained using the pulse pair method, the Rytov approximation and a wavelength of 564 nm. b) Image of one particle at different wavelengths together with a line cut of the RI along z. Scale bar: 500 nm. c) Averaged z-dependence of RI for the particles in a) acquired with the pulse pair method. Shaded areas correspond to two standard deviations. d) Averaged RI from NPs acquired with the slit scan method. e) Same NPs as in d) but reconstructed with Born approximation.

To validate the effect of the wavelength dependent k-space filling on our data, we perform a simple simulation. First, we save for each wavelength a binary mask in the k-space of points which our imaging system retrieves (Figure SI 8f). Then, in real space, we create a 3D array with the RI values of water and set a single voxel in the center to a RI value of 0.7, similar to Au. The pixel size in the experiment was 122 nm, which is close to 150 nm Au diameter. Therefore, we use the same pixel size, and perform analogously to the experiment a 3D FFT, remove the frequencies we would also loose in the experiment (missing cone), and perform the inverse FFT. The results are shown in Figure SI 8g. In contrast to the real data, the spectral missing cone problem causes a increase towards higher wavelengths. Further, it has a symmetric point spread function. Thus, we can conclude that (i) the decrease in the RI with higher wavelengths is due to the material and (ii) the artefact of a larger RI than water with higher wavelengths is due to the Rytov approximation.

References

1. Antoine Monmayrant, Sébastien J. Weber, B. C. A newcomer's guide to ultrashort pulse shaping and characterization. *J. Phys. B At. Mol. Opt. Phys.* **43**, 103001 (2011).
2. Lepetit, L., Chériaux, G. & Joffre, M. Linear techniques of phase measurement by femtosecond spectral interferometry for applications in spectroscopy. *J. Opt. Soc. Am. B* **12**, 2467–2474 (1995).
3. Sung, Y. *et al.* Optical diffraction tomography for high resolution live cell imaging. *Opt. Express* **17**, 266–277 (2009).
4. Lim, J. *et al.* Comparative study of iterative reconstruction algorithms for missing cone problems in optical diffraction tomography. *Opt. Express* **23**, 16933–16948 (2015).
5. Saba, A., Gigli, C., Ayoub, A. B. & Psaltis, D. Physics-informed neural networks for diffraction tomography. *Adv. Photonics* **4**, 066001–066001 (2022).
6. Lim, J., Ayoub, A. B., Antoine, E. E. & Psaltis, D. High-fidelity optical diffraction tomography of multiple scattering samples. *Light Sci. Appl.* **8**, 2047–7538 (2019).
7. Lauer, V. New approach to optical diffraction tomography yielding a vector equation of diffraction tomography and a novel tomographic microscope. *J. Microsc.* **205**, 165–176 (2002).
8. Scaffardi, L. B., Pellegri, N., De Sanctis, O. & Tocho, J. O. Sizing gold nanoparticles by optical extinction spectroscopy. *Nanotechnology* **16**, 158–163 (2005).
9. Fan, S., Smith-Dryden, S., Li, G. & Saleh, B. Reconstructing complex refractive-index of multiply-scattering media by use of iterative optical diffraction tomography. *Opt. Express* **28**, 6846–6858 (2020).
10. Zhou, K. C. & Horstmeyer, R. Diffraction tomography with a deep image prior. *Opt. Express* **28**, 12872–12896 (2020).
