# Supplementary material for: Ultrabroadband Optical Diffraction Tomography
Source: ACS Photonics. 2024 Aug 27;11(9):3680–7. doi: 10.1021/acsphotonics.4c00797 (PMC11413850; doi:10.1021/acsphotonics.4c00797)
Supplement: Supplementary file 2 — ph4c00797_si_002.pdf [file ph4c00797_si_002.pdf]

TABLE OF CONTENTS GRAPHIC:

Ultra-broadband Optical Diffraction Tomography

Martin Hörmann[1], Franco V. A. Camargo[2], Niek F. van Hulst[3,4], Giulio Cerullo[1,2], and Matz Liebel[3,5*]

[1]Dipartimento di Fisica, Politecnico di Milano, Piazza L. da Vinci 32, 20133 Milano, Italy

[2]Istituto di Fotonica e Nanotecnologie-CNR, Piazza L. da Vinci 32, 20133 Milano, Italy

[3]ICFO – Institut de Ciencies Fotoniques, The Barcelona Institute of Science and Technology, Av. Carl Friedrich Gauss, 3, 08860 Castelldefels, Barcelona, Spain

[4]ICREA – Institució Catalana de Recerca i Estudis Avançats, Passeig Lluís Companys 23, 08010 Barcelona

[5]Department of Physics and Astronomy, Vrije Universiteit Amsterdam, De Boelelaan 1081, Amsterdam, 1081 HV, The Netherlands

**email: m.liebel@vu.nl*

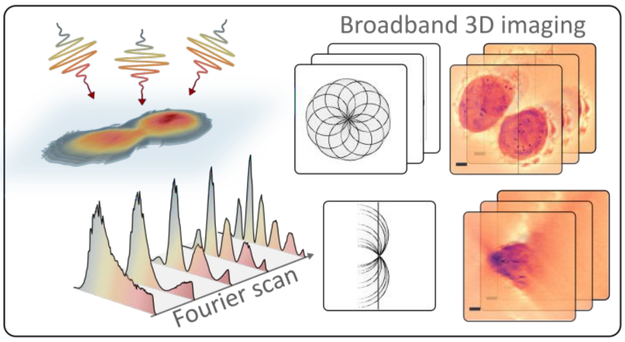

Ultrabroadband hyperspectral optical diffraction tomography for biological and functional imaging.
